# From Pregnancy to Breastfeeding: The Role of Maternal Exercise in Preventing Childhood Obesity

**DOI:** 10.3390/nu17040660

**Published:** 2025-02-12

**Authors:** Valeria Calcaterra, Hellas Cena, Agnese Pirazzi, Francesca Sottotetti, Erika Cordaro, Caterina Cavallo, Chiara Milanta, Dana El Masri, Maria Vittoria Conti, Matteo Vandoni, Gianvincenzo Zuccotti

**Affiliations:** 1 Department of Internal Medicine and Therapeutics, University of Pavia, 27100 Pavia, Italy; valeria.calcaterra@unipv.it (V.C.); erika.cordaro01@universitadipavia.it (E.C.); 2 Pediatric Department, Buzzi Children’s Hospital, 20154 Milano, Italy; chiara.milanta@unimi.it (C.M.); gianvincenzo.zuccotti@unimi.it (G.Z.); 3 Laboratory of Dietetics and Clinical Nutrition, Department of Public Health, Experimental and Forensic Medicine, University of Pavia, 27100 Pavia, Italy; hellas.cena@unipv.it (H.C.); francesca.sottotetti@unipv.it (F.S.); dana.elmasri01@universitadipavia.it (D.E.M.); mariavittoria.conti@unipv.it (M.V.C.); 4 Clinical Nutrition and Dietetics Unit, ICS Maugeri IRCCS, 27100 Pavia, Italy; 5 Laboratory of Adapted Motor Activity (LAMA), Department of Public Health, Experimental Medicine and Forensic Science, University of Pavia, 27100 Pavia, Italy; agnese.pirazzi01@universitadipavia.it (A.P.); caterina.cavallo01@universitadipavia.it (C.C.); 6 Department of Biomedical and Clinical Science, University of Milano, 20157 Milano, Italy

**Keywords:** exercise, physical activity, maternal, pregnancy, breastfeeding, childhood obesity, pediatrics, children

## Abstract

Low adherence to healthy lifestyle behaviors during pregnancy and lactation is strongly associated with a higher risk of childhood obesity. This narrative review aims to elucidate and summarize the pivotal role played by physical activity (PA) during pregnancy and breastfeeding, highlighting the potential mechanisms linking PA in these periods to the prevention of childhood obesity. Maternal exercise during pregnancy and breastfeeding significantly reduces the risk of childhood obesity by enhancing fetal metabolism, supporting healthy maternal weight management, and promoting improved breastfeeding practices. Pregnancy and the postpartum period represent critical windows for implementing preventive strategies that benefit both the mother and child. Encouraging an active lifestyle during pregnancy and breastfeeding is a vital public health strategy with extensive benefits. Healthcare professionals play a crucial role in creating supportive environments and providing tailored guidance to empower mothers to engage in regular PA. This approach not only enhances individual health outcomes but also contributes to the broader goal of fostering healthier communities.

## 1. Introduction

Childhood obesity is a worldwide critical health concern that is steadily increasing, leading to adverse short- and long-term health consequences [1,2]. According to the World Health Organization (WHO), overweight was reported to affect 37 million children under the age of 5 years in 2022 and 390 million children and adolescents aged 5 to 9 years, of which 160 million were affected by obesity [2].

Regional estimates differ across countries worldwide, yet all show constantly rising trends. Data analyzed by the World Obesity Federation suggest a further surge in the prevalence of overweight and obesity, estimating 206 million global cases in children and adolescents by 2025, with up to 254 million by 2030 [1].

**Figure 1:**
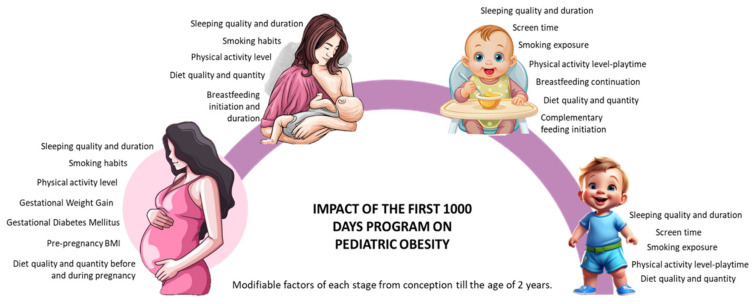
Impact of the first 1000 days program on pediatric obesity: modifiable factors at each stage, from conception to the age of 2 years, are synthesized [4,6,8].

The first 1000 days of life, spanning from conception to the child’s second birthday, represent a critical window for shaping long-term health outcomes, including the risk of pediatric obesity [3]. During this period, children’s growth and development are profoundly influenced by modifiable maternal and infant factors, such as maternal nutrition, pre-pregnancy body mass index (BMI), total gestational weight gain (GWG), physical activity (PA), breastfeeding practices in terms of duration and quality, infant birthweight, as well as broader environmental exposures [4]. These early influences are crucial as they set the fetal metabolic programming and can impact a child’s health, not only during childhood but also later in life during adulthood [5]. Research has indicated that low adherence to healthy lifestyle behaviors during pregnancy and lactation is strongly associated with higher risks of childhood obesity and abdominal adiposity, and that interventions targeting pregnancy and extending through the lactation phase further amplify the preventive potential of this critical period [6]. In Figure 1, we have schematized the modifiable factors at each stage, from conception to the age of 2 years, that influence the impact of the first 1000 days program on pediatric obesity.

During pregnancy, moderate and safe PA was found to positively affect the physiological and psychological status of the mother, which can indirectly influence the health status of the developing baby and the newborn’s weight outcome [7]. Maternal PA can also impact children’s weight status and childhood obesity through different factors. It has been found to reduce the likelihood of excessive GWG and gestational diabetes mellitus (GDM), both key contributors to childhood obesity [8]. A systematic review by Chen et al. demonstrated that PA during pregnancy is associated with a 17% reduction in the risk of large-for-gestational-age infants and a 55% reduction among mothers with pre-pregnancy obesity [9]. The intrauterine environment undergoes several metabolic changes when engaging in PA [2,3]. Some studies have found that altering the intrauterine and fetal environment might influence the gene expression that regulates the appetite and metabolism of the newborn [2].

During breastfeeding, PA was reported to promote the infant with healthy metabolic programming, regulate appetite, and support healthy weight gain. Prolonged breastfeeding duration, particularly when exclusive, has been linked to a lower likelihood of rapid weight gain in infants, with protective effects extending into later childhood [8,10]. PA appears to enhance the beneficial effects of breastfeeding by influencing breastmilk composition. Specifically, studies on the breastmilk of physically active mothers have reported an improved nutritional composition, which appears to positively influence infant metabolism and growth, while also reducing the risk of overweight and obesity [7]. Additionally, exclusive breastfeeding has been shown to protect against high body fat mass and obesity in later childhood, as reported by Jian Ma et al. [10]. Such findings underscore the importance of integrated approaches combining maternal physical activity, optimized breastfeeding practices, and early nutritional guidance to combat pediatric obesity effectively.

Despite a growing number of studies, the consistency of the findings is often influenced by the diversity of the investigated factors across the literature. However, research in the field of pediatric obesity, and PA during pregnancy and breastfeeding, underscores the potential role of these critical periods as strategic windows for obesity prevention [2,6].

This narrative review aims to elucidate and summarize the pivotal role of PA during pregnancy and breastfeeding, highlighting the potential mechanisms linking PA in these periods to the prevention of childhood obesity.

## 2. Methods

This narrative review [11] synthesized the current literature on the impact of maternal exercise during pregnancy and breastfeeding on childhood obesity. The aim was to provide a comprehensive qualitative analysis rather than a systematic or quantitative meta-analysis. Specific inclusion and exclusion criteria were applied to ensure the relevance and quality of the included studies. The inclusion criteria were articles written in English, publications within the last 20 years to ensure contemporary relevance, meta-analyses, clinical trials, and review articles focusing on the topic of maternal exercise and its impact on childhood obesity, and studies exploring related topics, including GWG, birthweight, and human breast milk (HBM) composition. Exclusion criteria were case reports and series due to their limited generalizability, studies unrelated to maternal physical activity, breastfeeding, or their combined effects on childhood obesity.

The literature search was conducted using the electronic databases PubMed and Web of Science. Keywords were used individually and in combination, encompassing terms such as physical exercise (PE), physical activity, maternal exercise, maternal lifestyle, pregnancy, lactation, breastmilk, breastfeeding, pediatric obesity, pediatric overweight, childhood obesity, childhood overweight, BMI, and birthweight. Key data were extracted from the included articles, focusing on study type, sample size, interventions, outcomes related to maternal PA and breastfeeding, and their impact on childhood obesity.

The initial search yielded 2047 articles. Duplicate records were removed before screening (n = 1165). Titles and abstracts were screened for relevance (n = 882), resulting in 210 full-text studies selected for detailed evaluation. After applying the inclusion criteria, 106 articles were included in the final review. To ensure comprehensive coverage, the reference lists of the selected articles were reviewed to identify additional relevant studies (n = 38). The paper used for the general part of the manuscript was also included (n = 21).

**Figure 2:**
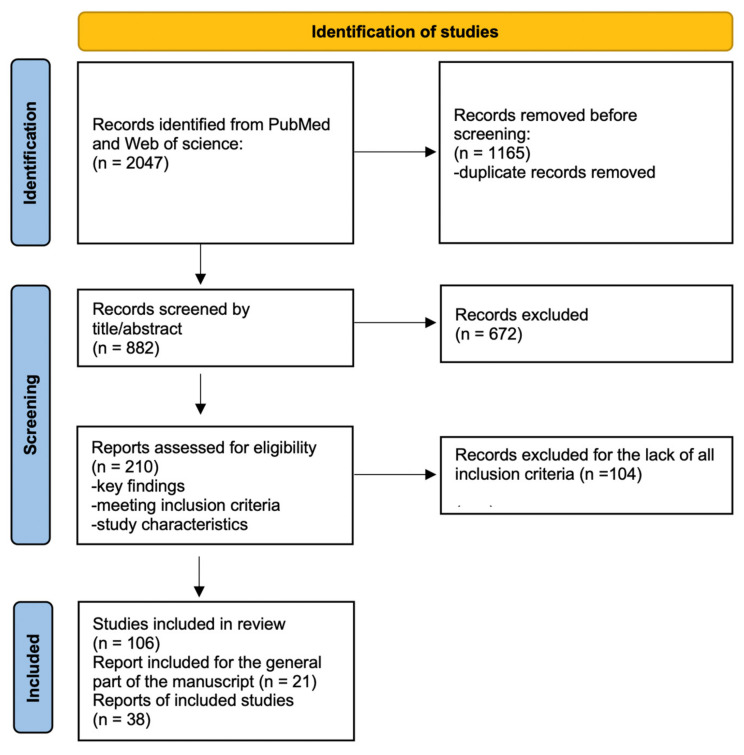
Flowchart of the search and selection of articles process.

A flowchart summarizing the search and selection process is provided in Figure 2. This diagram illustrates the progression from the initial search results to the final included articles, highlighting the exclusion steps at each stage.

## 3. Maternal Exercise During Perinatal Period

### 3.1. Exercise Recommendations Based on Gestational Period

Pregnancy is characterized by significant physical and psychological changes, making women particularly susceptible to mental and physical health challenges [12]. Scientific evidence underscores that regular PA during pregnancy offers numerous benefits [13]. To optimize maternal and infant health, the World Health Organization (WHO) recommends that pregnant and postpartum women engage in at least 150 min of moderate-intensity aerobic PA weekly, complemented by muscle-strengthening and gentle stretching exercises [14,15].

Despite these recommendations, widespread misconceptions about the risks of exercise during the perinatal period hinder participation in PA programs during pregnancy and in the postpartum period [16]. Contrary to these beliefs, evidence demonstrates that PA is not associated with increased risks of miscarriage, preterm birth, low neonatal birthweight, or perinatal mortality [17,18].

Integrating PA during pregnancy and breastfeeding provides substantial benefits for both mothers and infants. Specifically, improvement or maintenance in this period is enhanced by PE, a subcategory of PA. PE is planned, structured, repetitive, and regulates maternal stress hormones, alleviating symptoms of depression and anxiety [19]. Similarly, Tiemeyer et al. reported that PE improves maternal self-image, self-confidence, body image, and emotional well-being [20]. Structured and repetitive movement also positively affects weight management, reduces the onset of GDM, and mitigates cardiovascular issues such as high blood pressure and pre-eclampsia [21,22].

Fetal benefits are equally significant. Álvarez-Bueno et al. showed that PE promotes placental growth and function, enhancing fetal development by ensuring sufficient oxygen and nutrient supply [23]. Supporting this, Hoirisch-Clapauch et al. highlighted that umbilical cord function facilitates metabolic regulation and fetal growth, which correlates with maternal lifestyle [24]. Despite these benefits, many pregnant women decrease their PA and PE levels during pregnancy, often failing to meet the recommended guidelines [25]. To corroborate, the WHO confirms that PA promotes maternal and fetal health without compromising safety [26]. However, recommendations should be personalized based on medical history and pregnancy conditions [18].

PA performed under professional supervision reduces the risks of GDM, pre-eclampsia, preterm birth, and excessive GWG while improving insulin sensitivity [18,22]. Both home-based and supervised exercise programs are effective options [27,28]. PA improves cardiovascular function, enhancing cardiorespiratory fitness and reducing heart rate frequency [29]. Ferrari et al. found that women participating in PE programs during pregnancy experience better stamina, muscle strength, and energy levels [30].

Pregnant women can engage in three main types of PE: aerobic, resistance, and stretching exercises [31]. Aerobic exercises enhance cardiovascular health, while resistance training builds muscle strength, particularly in the pelvic floor, reducing urinary incontinence and improving postpartum recovery. Barakat et al. demonstrated that moderate aerobic exercise performed three times per week until 38–39 weeks reduces excessive GWG and GDM risk [32]. Stretching exercises, which improve flexibility and reduce musculoskeletal tension, are particularly beneficial as pregnancy progresses [33,34]. Monitoring adverse symptoms during PE is essential to ensure safety for both the mother and fetus [35].

Exercise recommendations vary across trimesters. During the first trimester, moderate-intensity, low-impact exercises help to alleviate symptoms like nausea and fatigue [18,31]. Song et al. reported that moderate aerobic activities reduce unfavorable pregnancy outcomes [36]. Light resistance exercises and yoga can also enhance flexibility and tone [31]. In the second trimester, there are weight management benefits from a gradual increase in exercise intensity as the metabolism accelerates [37]. Swimming and stationary cycling are particularly beneficial for minimizing joint stress while providing cardiovascular benefits [38].

During the third trimester, low-impact activities like walking reduce sedentary time and prenatal anxiety [27,39]. Beetham et al. confirmed that vigorous exercise in healthy pregnancies during this period remains safe [40]. Perales et al. [39] suggested focusing on balance training to enhance postural stability, reduce fall risk, and improve core strength.

### 3.2. Effects of Exercise on Molecular and Epigenetic Mechanisms Related to Maternal Exercise

Maternal exercise induces significant biological changes at the molecular and epigenetic levels. At the molecular level, PE modulates metabolic, inflammatory, and oxidative pathways, enhancing maternal health and fostering a favorable intrauterine environment [18]. Exercise also influences epigenetic modifications, including DNA methylation, histone changes, and microRNA (miRNA) expression. Franzago et al. demonstrated that these adaptations affect gene regulation in maternal and fetal tissues, improving fetal development and reducing chronic disease risk [41].

PE significantly impacts the endocrine system, modulating hormones like endorphins and cortisol. Endorphins enhance mood, while balanced cortisol levels mitigate chronic stress, benefiting neurodevelopment [42,43]. PE also improves insulin sensitivity, lowering maternal GDM risk and reducing the child’s future risk of metabolic disorders [21,44]. Xie et al. highlighted the importance of PE during the first trimester in preventing GDM [21].

PE reduces systemic inflammation by lowering C-reactive protein (CRP), a marker of cardiovascular strain and pregnancy complications like pre-eclampsia [45,46]. Improved endothelial function during exercise enhances blood flow and reduces vascular stress, supporting fetal development [45,47]. Additionally, PE mitigates oxidative stress by increasing antioxidant enzyme levels, protecting maternal and fetal cells from damage [48,49]. Hussain et al. reported that PE enhances mitochondrial function, optimizing nutrient and energy transfer to the fetus [49,50,51].

At an epigenetic level, PE targets genes associated with metabolism, growth, and stress response. DNA methylation modulates metabolic efficiency, while histone modifications influence gene expression [52,53,54]. Lycouid et al. found that miRNA modulation through PE supports placental angiogenesis, enhancing fetal growth and development [55]. Gomes Da Silva et al. demonstrated that these changes improve neonatal brain function and cognitive abilities [56]. Children of physically active mothers exhibit improved glucose regulation, lower body fat, and enhanced cardiovascular health compared to the offspring of sedentary mothers [57].

## 4. Impact of Maternal Exercise During Pregnancy on Weight Growth

### 4.1. Effects of Maternal Physical Activity During Pregnancy on Birthweight

Maternal PA during pregnancy provides significant benefits, including a reduction in the risk of developing GDM and excessive GWG. Recent studies have also explored its potential role in regulating birthweight, though findings remain mixed [58]. This narrative review includes trials conducted over the last five years, employing diverse methodologies to investigate the impact of PA on at-term newborns worldwide.

A randomized controlled trial (RCT) conducted in 2023 compared three groups: structured supervised exercise training (three times a week), motivational counseling on PA (seven sessions throughout pregnancy), and standard prenatal care. The results indicated no significant differences in GWG or birthweight among the groups [59]. Similarly, a study by Raper et al. recruited women aged 18–40 years with a BMI of 18.5 to 35 kg/m^2^ and assigned them to either moderate-intensity aerobic exercise (150 min per week) or low-intensity stretching and breathing sessions. The birthweight outcomes did not differ between the groups [60].

Concerning GDM, a fitness training program for Iranian women with low-risk pregnancies after in vitro fertilization was shown to lower the incidence of GDM without significant changes in birthweight [61]. In a separate study, Ding et al. evaluated 215 overweight or obese pregnant women, randomizing them into intervention and control groups. The intervention included personalized dietary and PA recommendations, supported by a monitoring tool and daily step goals (e.g., walking 6000 steps). Although the intervention group exhibited a lower GDM incidence (24.5% vs. 37.8% in controls), there were no significant differences in the macrosomia rates [62].

Further research by Yew et al. focused on women with GDM between 12 and 30 weeks of gestation. The participants were randomized to either standard care or an integrated program incorporating nutritional guidance, PA, and glucose monitoring. While the glucose levels and neonatal complications were significantly lower in the intervention group, the birthweight outcomes remained unchanged [63]. A similar study in 2023 involving 211 women with GDM also observed no differences in birthweight between groups, although birth length showed a marginally significant improvement in the intervention group [64].

In Taiwan, 92 overweight or obese women were randomized into standard prenatal care or an active health intervention. The results revealed that women in the intervention group had lower body weights before delivery and their infants had significantly lower birthweights compared to the controls [65]. Supporting these findings, the LIMIT trial demonstrated that dietary and lifestyle improvements significantly reduced the incidence of high birthweight among pregnant women with overweight or obesity [66].

A systematic review combining data from 2017 to 2020 with earlier analyses (1990–2017) found that pregnancy-related PA consistently reduced GWG, GDM risk, and other adverse outcomes. However, no statistically significant association was observed between PA and birthweight [67]. Another review examining high-risk pregnancies with bed rest recommendations highlighted significant variability in the relationship between activity restriction and birthweight. It concluded that even in cases of recommended bed rest, some level of PA might offer benefits if feasible [68]. The evidence supports that PA during pregnancy positively impacts maternal health by lowering GWG and GDM risks. However, its direct effects on birthweight remain less conclusive, likely influenced by various maternal and fetal factors. Further studies are needed to isolate the specific contributions of PA to birthweight outcomes, accounting for confounding variables such as maternal comorbidities and other health interventions [61,62,65].

Table 1 summarizes studies on the effect of maternal physical activity during pregnancy on birth weight.

### 4.2. Effects of Maternal Physical Activity During Pregnancy on Children’s BMI

The pregnancy and preconception periods are critical windows for implementing interventions to mitigate childhood obesity risks, with parental lifestyle behaviors serving as significant determinants [69].

A recent study using data from four European mother–offspring cohorts within the EndObesity Consortium, encompassing 31,200 families, analyzed various lifestyle patterns. The findings revealed that maternal sedentary behavior, poor diet quality, and parental smoking during pregnancy were strongly associated with higher child BMI Z-scores and a greater risk of childhood overweight and obesity at five years of age [69].

To evaluate the long-term impact of lifestyle interventions compared to standard prenatal care, the LIMIT study enrolled overweight or obese women and tracked their children from birth to ten years. Despite providing nutritional and lifestyle recommendations to the mothers, this study found no significant reduction in the risk of childhood obesity. Nearly 45% of children exhibited BMI Z-scores exceeding the 85th percentile by age ten, indicating a persistent risk of obesity into adolescence and adulthood [66].

Haby et al. analyzed data from children aged 2.5 years born to mothers who received either standard prenatal care or lifestyle support aimed at limiting prenatal weight gain. Their findings indicated that maternal BMI correlated with the children’s risk of overweight and obesity, yet no direct link was found between maternal participation in the intervention and children’s weight outcomes [70].

In Norway, Bjøntegaard et al. conducted a study involving 855 women randomly assigned to either standard prenatal care or an exercise intervention during pregnancy. The results suggested a strong correlation between maternal and child BMI, regardless of whether the mother engaged in PA during pregnancy [71].

A pooled analysis of two RCTs, including 1348 pregnant women assigned to either standard care or supervised moderate-intensity exercise (three sessions per week), found that PE during pregnancy reduced GWG, the risk of GDM, hypertension, and diabetes. Importantly, it also significantly decreased the likelihood of childhood overweight and obesity during the first year of life [72].

Other studies have explored the link between maternal PA during pregnancy and childhood BMI. For example, Mitanchez et al. identified early pregnancy GWG as a predictor of higher BMI and adiposity in offspring. This highlights PA as a potential strategy to mitigate excessive GWG, though adherence to PA programs remains a challenge for many women [73]. Additionally, another review found associations between healthy pregnancy factors and childhood obesity risk but did not isolate PA as an independent variable [74]. A systematic review and meta-analysis of 30 RCTs involving 16,137 women also concluded that PA during pregnancy had no significant effect on children’s BMI [75].

While PA during pregnancy is highly recommended for reducing GDM and GWG, its role in preventing childhood obesity remains unclear. The evidence suggests short-term benefits, such as a reduced risk of childhood overweight during the first year of life, but the long-term effects are inconsistent and influenced by confounding factors. Further research is necessary to establish the independent impact of maternal PA on childhood BMI trajectories, free from external biases.

Table 2 summarizes studies on the impact of maternal physical activity and lifestyle interventions during pregnancy on childhood BMI.

## 5. Impact of Maternal Exercise During Breastfeeding on Childhood Obesity

### 5.1. Effect of Physical Activity on Breastmilk

Breastfeeding significantly improves overall quality of life for both mothers and infants [76]. Exclusive breastfeeding, as recommended by the WHO for the first six months of life, provides significant protective effects against childhood obesity, high body fat mass, and certain non-communicable diseases (NCDs) later in life [77]. Additionally, it supports optimal growth, development, and respiratory health [76,77]. In this context, it is essential to analyze how PA can influence the composition of human breast milk (HBM) and the breastfeeding process in general. According to the American College of Obstetricians and Gynecologists (ACOG), regular aerobic exercise in breastfeeding women can enhance maternal cardiovascular fitness without impacting milk production, composition, or infant growth [78]. Breastfeeding women who engage in PA should ensure that they meet the adequate dietary and fluid intake requirements. In addition to the energy expenditure associated with any bodily movement, it is important to account for the approximately 450–500 kcal/day required for lactation [79]. Recommendations include breastfeeding before exercising, waiting at least one hour after exercising to breastfeed, or using previously expressed milk if the baby shows a need to breastfeed immediately after the mother exercises [80]. PE has shown to impact HBM even before the lactation period, during pregnancy. A study by Aparicio et al. involved pregnant women randomly assigned to either an exercise group (three 60 min sessions per week, combining aerobic and strength training) or a control group, starting from the 17th week of gestation until delivery [81]. This study evaluated the levels of pro-inflammatory and anti-inflammatory cytokines in the colostrum and mature milk. The findings revealed that the exercise program encouraged a less pro-inflammatory profile, especially in colostrum [81]. Moreover, the frequency and intensity of PA during pregnancy may influence breastfeeding duration. A prospective cohort study was conducted to examine the relationship between antenatal PA and the duration of breastfeeding among 1715 women who were recruited between 24 and 28 weeks of gestation and followed for 12 months postpartum. PA data were collected using a questionnaire. The results showed that, at 12 months, 71.8% of mothers were still engaged in breastfeeding. Notably, mothers with higher levels of PA were shown to be more committed to breastfeeding for 12 months compared to those with lower PA levels (odds ratio, OR = 1.71 vs. 1.38) [82].

The clinical trial conducted by Be’er et al. examined the macronutrient composition and energy content of HBM from 31 mothers who expressed milk on two consecutive days (one day before and after engaging in PA and one control day without any PA).

The results indicated that moderate-to-high-intensity PA had no significant effect on the levels of macronutrients (fat, carbohydrates, protein) or the energy content of the milk. Additionally, the volume of milk expressed remained unchanged [83].

Concerning specific elements within HBM, adiponectin plays an important role in glucose and fat metabolism. It is supposed to support infant metabolic development and may offer protection against rapid weight gain during early childhood [84]. In this regard, a randomized cross-over study investigated the effect of resistance exercise on adiponectin concentrations in HBM [85]. Participants, exclusively breastfeeding mothers, underwent three experimental conditions: moderate-intensity continuous training (MICT), high-intensity interval training (HIIT), and no activity (REST). The adiponectin concentrations in HBM were measured at four time points. The results showed a significant increase in adiponectin concentrations 1 h after HIIT, with a 0.9 μg/L change (95% CI: 0.3 to 1.5) compared to the REST condition (*p* = 0.025) [85].

Moreover, an important type of lipokine known as 12,13-dihydroxy-9Z-octadecenoic acid (12,13-diHOME) plays a role in regulating fuel uptake and thermogenesis in brown adipose tissue. This lipokine was recently identified in HBM, and its abundance is inversely correlated with infant adiposity, suggesting that variations in HBM composition may be linked to early obesity risk. Plasma concentrations of 12,13-diHOME acutely increase following PE; however, some studies suggest that these effects are transient. Similarly, 12,13-diHOME concentrations in HBM rise acutely after PE in most women, although it remains unclear whether this effect plays a causal role in limiting rapid weight gain in infants [86].

Additionally, human milk oligosaccharides (HMOs) are known to have a positive effect on the health outcomes of newborns [87]. HMO concentrations in the HBM exhibit significant intra- and inter-individual variability, which can be influenced by a range of maternal factors [88]. However, the effect of PA on the HMOs content in HBM has not been thoroughly investigated. A recent study by Biddulph and colleagues sought to examine the potential impact of short-term maternal variables on HMO profiles in mature HBM [89]. Milk samples were collected at 3–4 months postpartum from 101 Australian women. The results indicated that maternal postpartum PA levels had a significant effect on HMO composition (*p* > 0.05) [89].

Despite the increasing interest in microbiota research, there is currently a lack of studies specifically examining the effects of maternal PA on the composition of HBM microbiota. Nonetheless, several studies have documented the positive effects of PA on gut microbiota, highlighting a rise in bacterial diversity and richness [90]. Regarding the relation between the mammary glands and the mother’s intestinal tract, it is plausible that gut bacteria may migrate to the mammary gland via an endogenous pathway known as the active migration theory [91]. Consequently, the modulation of the maternal gut microbiota through PA or PE holds the potential to indirectly influence the composition of microbiota in HBM [91].

Although the positive effects of PE on breastfeeding have been extensively studied and highlighted, it is noteworthy that PA level often decreases during the lactation period. In this context, the findings of Cabrera-Domínguez et al. are particularly insightful [92]. This study aimed to assess various factors, including PA, in a cohort of women during both pregnancy and the postpartum breastfeeding period (≤6 months post-delivery) using a questionnaire. A significant reduction in the daily PA level was observed at the end of pregnancy and during the lactation period. Notably, a decrease in the total activity level was specifically associated with breastfeeding (*p* = 0.016). Additionally, women in the breastfeeding group reported lower levels of social support compared to the gestational period [92]. These findings underscore the potential negative impact of reduced sleep quality, diminished PA, and lower social support on women’s health and overall well-being during lactation.

Table 3 summarizes studies on the impact of maternal exercise during breastfeeding on childhood obesity.

### 5.2. Association Between Breastfeeding and Childhood Obesity

Breastfeeding has been widely recognized to play a pivotal role in the optimal growth of the newborn, exerting an influence both on short-term and long-term health [93]. The composition of HBM is dynamic and changes across the lactation stages, closely following the needs of the infant. It provides adequate energy and bioactive factors that promote somatic growth, regulate postnatal intestinal functions, boost immunity, and support brain development [94] as well as metabolic development [95,96]. Moreover, HBM and formula milk present different compositions of fat, protein, vitamin, and mineral content [94], with a higher cholesterol (due to the presence of fat globule membrane in formula milk, as it primarily derives from bovine milk [97]) and protein content in formula milk than in HBM. Research revealed a strong correlation between increased protein intake during infancy, weight gain, and the likelihood of developing obesity [98,99]. Therefore, the WHO guidelines recommend exclusive breastfeeding at least for the first six months of life and until the child is two years old or older with the concomitant introduction of complementary foods to achieve optimal development [100]. Despite that, according to statistical analysis, only 44% of infants are exclusively breastfed globally [101], with data reported by the Higher Institute of Health in Italy for the year 2022 confirming this trend (46.7% of children aged 2–5 months exclusively breastfed), characterized by a lower rate in the South compared to the Center-North [102].

It is noteworthy that breastfeeding during the first 1000 days of life has been demonstrated to reduce the risk of developing pediatric obesity throughout the modulation of risk factors usually involved [103]. A meta-analysis revealed, in fact, that breastfeeding is linked to a 13% decrease in the likelihood of overweight or obesity [104]. Azad et al. reported that breastfeeding is inversely associated with weight gain velocity and BMI in a dose-dependent manner [105]. A cohort study conducted by Ong YY et al. reported that children breastfed for less than four months had increased BMI at age 6 in comparison with those breastfed for over four months [106]. Similarly, in the United States, a study examined children aged 4 to 8 and discovered a dose–response relationship between breastfeeding and the likelihood of developing pediatric obesity, with a reduction in the risk of 60% for those who were breastfed for 6 months compared to non-breastfed counterparts [107]. A longitudinal study also showed that each additional month of exclusive breastfeeding was associated with a 1% decrease in BMI and a 2% reduction in fat mass at 6 years of age [108]. Moreover, it has been demonstrated that adults who were breastfed had lower rates of overweight and obesity, type 2 diabetes [109], and hypertension [110,111] than those fed infant formula, thus confirming the importance of breastfeeding for long-term health.

The mechanisms through which breastfeeding seems to counteract the risk of developing obesity are different and are based on the influence exerted by the bioactive components introduced as well as the modulation of gene expression exerted by HBM itself [112,113].

Firstly, HBM is the most enriched source of miRNAs among all human body fluids [114]. Abbas et al. demonstrated the association between exosomal milk miRNAs and the regulation of adipogenesis [115]. Different studies reported that miRNA-148a-3p, a crucial component of milk exosomes [108,116], directly targets DNA methyl-transferase 1 (DNMT1) [117,118], while the Targetscan Human 8.0 database revealed that miRNAs contained in HBM exosomes such as miRNA-30-5p, miRNA-21-5p, and miRNA-155-5p all target FTO [119]. Their action results in the regulation of adipogenesis and protection against early adipogenic lineage commitment.

Also, HBM contains leptin, adiponectin, and ghrelin, hormones involved in the regulation of appetite and energy balance that, therefore, play a key role in metabolic health [120,121]. Ghrelin, for example, was discovered to have an inverse correlation with weight gain among breastfed children compared to formula-fed counterparts [120,121]. Adiponectin in HBM is also associated with a lower risk of childhood overweight [122]. In general, the plasmatic concentration of these hormones appears to be further enhanced by the effect of HBM on the genetic profile of the newborn. The cross-sectional study conducted by Wijnands KP et al. in 17-month-old infants revealed a negative correlation between the duration of breastfeeding and the LEP gene methylation level [123]. These findings were also confirmed by Sherwood et al., whose research in children aged 10 who were breastfed for longer demonstrated a decreased LEP gene methylation level [124,125]. The less LEP gene is methylated the more its expression is enhanced, thus suppressing hunger and helping to prevent the development of pediatric obesity. The epigenetic modulation exerted by breastfeeding seems to also affect other genes involved in energy metabolism, such as fat mass and obesity-associated (FTO) gene, Retinoid X Receptor Alpha (RXRA), and human peroxisome proliferator-activated receptor gamma (PPAR-γ) gene. HBM has been reported to alter the DNA methylation of the FTO gene, well known for its variance among individuals and its strong correlation with obesity [126]. In fact, Cheshmeh et al. found that the FTO gene expression level was significantly lower in breastfed infants compared to formula-fed and mixed-fed infants [127]. Kanders et al. demonstrated breastfeeding moderating action in the association between FTO rs9939609 polymorphism (defined as an obesity-related polymorphism because of its role in increasing BMI and adiposity [128,129]) and children’s BMI [130], as also reported by Wu YY et al., whose research demonstrated that five months of exclusive breastfeeding attenuated the increase in BMI and delayed the timing of the adiposity peak among FTO rs9939609 variant carriers [131]. However, further evidence is still needed as, for example, data reported from a study conducted by Dedoussis et al. revealed a correlation between breastfeeding and rs9939609 and rs17817449 FTO variants in two Greek cohorts, but not in a British cohort [132].

Pauwels et al. demonstrated that infants breastfed for 6 months had significantly lower RXRA gene methylation levels compared to non-breastfed infants, leading to inversely linked HBM to RXRA gene methylation levels [133].

Finally, the study conducted by Verier et al. [134] showed that among children carrying the high-risk obesity-related phenotype Pro12Ala of the PPAR-γ gene BMI, waist circumference and skinfold thickness all decreased with an increased duration of breastfeeding compared to children who were formula-fed, thus suggesting that breastfeeding might modulate PPAR-γ gene transcriptional activity among individuals carrying the Pro12Ala allele. As reported before, more investigations are needed as no influence on weight gain was detected in children without PPAR-γ gene Pro12Ala polymorphism in both breastfed and formula-fed children [134].

It could be possible that breastfeeding exerts its preventing function on childhood obesity mostly in children with specific genetic backgrounds. However, the benefits related to breastfeeding are not only related to its epigenetic effects.

Breastfed infants were reported to have a better ability to regulate their food intake compared to bottle-fed infants [135], which may show that they have a poor self-regulation of intake and reduced prolonged satiety both in the newborn period and in later childhood [136]. A possible explanation is that breastfeeding allows the infant to have more control over the volume of milk swallowed in every feed, while, in bottle-feeding, the mother has more control and leads the baby to consume more, for example, through encouraging it to finish the bottle or adding feeds to increase the infant’s sleep period [137]. Moreover, breastfed infants are more likely to consume fruits and vegetables and to be less picky, having, in general, a healthier diet compared to their formula-fed counterparts [138,139,140]. This is because of their exposure to different flavors through their mother’s diet before and directly through the consumption of solid foods [141,142]. Moreover, it was then reported that breastfed children who were breastfed for the first 6 months of life were less likely to show a desire to drink sugar products [121]. Interestingly, infants’ circadian rhythms, that after birth are still immature [143], are also influenced by breastfeeding.

It was reported that HBM contains high levels of tryptophan (TRP) [144] and that TRP acts as a cue in the regulation of the biological rhythms in the BF breastfed infant [145]. Lodemore et al. observed that circadian rhythmicity in body temperature appeared earlier in BF breastfed infants compared to formula-fed counterparts [146].

Finally, breastfeeding is the main factor that constitutes infant intestinal microbiota [147]. It was demonstrated that breastfed infants have higher levels of Bifidobacterium compared to formula-fed infants [148,149] thanks to skin-to-skin contact immediately after birth, with the first feedings of colostrum, and with breastfeeding. HBM contains probiotics that colonize the infant gut [150].

It is important to note that these benefits are directly related to the mother’s diet and the mother’s BMI, which influence HBM composition. Research has shown that HBM from obese mothers with obesity has significant differences in the concentration of metabolites with respect to that of normal-weight mothers; this may affect infant postnatal growth and long-term health [151].

Therefore, although the true potential of breastfeeding remains partially unclear and needs further investigation, all these results, taken together, allow us to say that HBM provides the baby with enormous benefits. Understanding the complex interactions in which breastfeeding is involved and studying the epigenetic mechanisms affected by the introduction of HBM will provide valuable insights into the prevention of childhood obesity, supporting also the development of personalized approaches.

Table 4 summarizes studies on the role of breastfeeding in weight control.

## 6. Physical Intervention Strategies During Pregnancy and Breastfeeding

Various studies have explored strategies to promote PA during pregnancy and breastfeeding, emphasizing tailored guidance from specialists within prenatal and postnatal care. Incorporating PA into these periods not only enhances maternal health but also influences HBM composition without affecting milk supply volumes [153]. Studies suggest that the HBM of physically active mothers has improved nutritional properties, which positively impact infant metabolism and growth while reducing the risk of overweight and obesity [86,120].

Despite the established benefits of PA, approximately 75% of pregnant women fail to meet the recommended guidelines, and sedentary behaviors are expected to increase in the coming years [15,16]. Pregnancy introduces numerous physical and psychological changes, some of which may act as barriers to PA. For example, the progressive increase in body weight during pregnancy elevates energy demands and makes weight-bearing activities more physically taxing [18].

**Figure 3:**
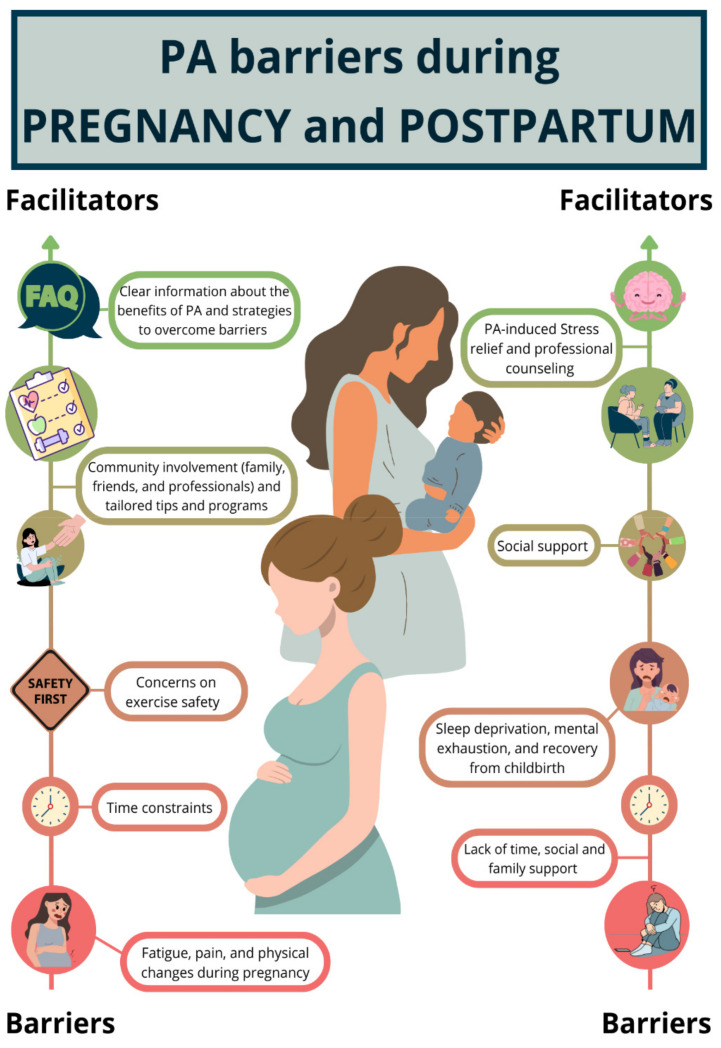
Graphical representations of facilitators and barriers of physical activity (PA) practice based on prior qualitative studies [160,161,162,163]. PA = physical activity.

Similarly, the postpartum and breastfeeding periods present unique challenges. Women often prioritize their newborns’ needs, leading to reduced personal time and increased sedentary behavior associated with breastfeeding [154,155,156]. Further examples of factors that act as barriers and facilitators of PA practice are presented in Figure 3.

To promote a healthier lifestyle during these periods, interventions must accommodate maternal needs and conditions.

### 6.1. Tailoring Physical Activity Interventions

To effectively implement PA strategies, women’s previous fitness levels should be considered. Active pregnant women can safely engage in moderate-intensity exercise, while sedentary women should begin with light-intensity activities and gradually increase intensity over time. During pregnancy and breastfeeding, PA programs can include aerobic, strength, and mobility exercises customized to maternal conditions. These exercises not only improve cardiovascular fitness, muscle mass, and flexibility but also reduce the risk of gestational complications and optimize postpartum recovery.

Hawkins et al. demonstrated the efficacy of tailored interventions in improving PA levels during the first trimester of pregnancy. Women in the intervention group were, on average, 93 min per week more physically active than the controls. Participants were offered various activity options, such as dancing, walking, and yard work, and received personalized tips to overcome barriers and adopt an active lifestyle [157].

Similarly, a study by Lewis et al. [14] highlighted that postpartum women participating in an exercise intervention maintained or increased their PA levels at 6-month and 9-month follow-ups compared to wellness and usual care groups. The intervention involved telephone sessions with health educators who provided heart-rate-based intensity guidelines, activity durations, and specific exercise types [158].

### 6.2. Challenges in Physical Activity Interventions

Not all PA interventions yield consistent success. A systematic review by Peralta et al. [159] reported contrasting results among group-based interventions targeting postpartum women with children aged 0–5 years. The authors found that while home-based and remotely supervised exercise protocols achieved higher engagement rates, interventions requiring on-site attendance were less effective. Furthermore, interventions solely focusing on PA were generally more successful than those attempting to address multiple health behaviors, such as diet or sleep habits [159].

Another systematic review on postpartum women emphasized the importance of PA interventions in promoting weight loss and reducing the risk of comorbidities. However, this review concluded that the most effective intervention types for postpartum weight loss remain unclear [156].

The evidence supports the value of tailored PA interventions during pregnancy and breastfeeding for improving maternal and infant health outcomes. Personalized approaches, including home-based and remote programs, appear to offer greater success in engaging women in PA. Further research is needed to identify the most effective intervention strategies for promoting sustained PA and long-term health benefits during these critical periods, Figure 3.

Details of the mentioned PA interventions are summarized in Table 5.

## 7. Limits

This review has several limitations that must be acknowledged. As a narrative review, it provides a qualitative, non-systematic analysis of the existing literature [11]. The lack of standardized guidelines for conducting narrative reviews may introduce selection bias, as the studies included were limited to those indexed in PubMed and Web of Science. Consequently, relevant studies available in other databases or search engines may have been excluded. This limitation was partially mitigated by screening reference lists and cross-referencing related studies.

Another significant limitation is the paucity of research directly examining the impact of maternal exercise on the risk of childhood obesity. This gap is largely attributable to confounding factors, making it challenging to isolate the effects of PA. Further studies are required to explore this relationship while rigorously controlling for variables such as maternal comorbidities and the influence of non-exercise-related interventions.

Similarly, the relationship between PA and birthweight remains unclear. We selected studies that considered the potential effect of PA on birthweight, even though these studies are highly heterogeneous. It cannot be excluded that these parameters are strongly influenced by multiple variables, including maternal health conditions and other interventions that do not specifically target PA. Further research is necessary to eliminate these biases and clarify the role of PA in influencing birthweight outcomes.

Specifically, randomized controlled trials are needed to evaluate the effect of PA on birthweight and/or children’s BMI during pregnancy in normal-weight women without any comorbidities, apart from sedentary behavior.

Lastly, while exercise during the postpartum period is recognized as beneficial for promoting weight loss and reducing the risk of comorbidities, the optimal type, intensity, and duration of interventions for achieving postpartum weight loss remain undefined. Further investigation into this area could facilitate the development of tailored intervention strategies to maximize health benefits for mothers.

To conclude, we did not discuss the role of maternal lifestyle in shaping the subsequent lifestyle of children. A mother who is physically active during pregnancy and breastfeeding encourages the child to adopt an active lifestyle in the early years of life. This represents an additional factor that could be considered in preventing childhood obesity.

## 8. Conclusions

Maternal exercise during pregnancy and breastfeeding seems to offer benefits, reducing the risk of childhood obesity by improving fetal metabolism, promoting healthy maternal weight, and enhancing breastfeeding practices. The most recent literature underscores pregnancy and the postpartum period as critical windows for implementing preventive strategies that positively impact the health of both the mother and child.

PA during breastfeeding has been shown to improve HBM composition, enhancing the concentration of key bioactive components such as adiponectin and lipokines, which are beneficial for obesity prevention [85,86]. Furthermore, PA supports prolonged breastfeeding duration and overall maternal and infant well-being. However, many mothers reduce their PA levels during the postpartum period [92], which diminishes these protective effects. This highlights the need for targeted efforts to support and encourage personalized PA interventions during pregnancy and breastfeeding.

Despite the limitations of our review, the literature data support that encouraging an active lifestyle during these periods, as a key component of primary prevention strategies, could yield significant benefits for both mothers and their children. By fostering supportive environments and providing tailored guidance, healthcare professionals can help mothers harness the protective benefits of PA, contributing to healthier outcomes for the entire population.

## Figures and Tables

**Table 1:** Summary of studies investigating the impact of maternal physical activity during pregnancy on birthweight.

Author	Type of Study	Location	Sample	Study Purpose	Intervention Strategy	Adherence Rate (%)	Main Result
Roland et al. [59]	Randomized controlled trial	Denmark	A total of 219 pregnant women.	The effect of physical activity on GWG and neonatal outcome	Exercise 3 times/week vs. counseling 7 times/pregnancy vs. standard care	81%	GWG at GA 40 weeks did not differ, neither did obstetric nor neonatal outcomes, including birthweight.
Raper et al. [60]	Comparative quasi-experimental clinical trial	Iran	A total of 170 pregnant women who underwent IVF.	The effect of physical activity on the improvement in maternal and fetal health in women who underwent IVF.	Exercise training (walking, aerobic, strength-conditioning, and relaxation exercises) vs. no training	NA	A significantly lower GDM and pre-eclampsia rate was found in the intervened IVF-pregnant women. However, there was no significant difference in other negative maternal and neonatal outcomes between the two groups, including birthweight.
Charkamyani F et al. [61]	Randomized controlled trial	Beijing (China)	A total of 215 obese or overweight pregnant women.	The influence of a WeChat-based dietary and exercise intervention on GDM prevention in overweight/obese pregnant women.	General advice vs. personalized program, including We Chat-based dietary and exercise intervention	NA	WeChat-assisted dietary and exercise intervention was effective in reducing the occurrence of GDM and excessive GWG in overweight/obese pregnant women. However, the incidence of macrosomia was not significantly lower in the intervention group than in the control group.
Ding et al. [62]	Secondary data analysis from prospective randomized study (ENHANCED by Mom) focused on the influence of exercise type on infant outcomes throughout pregnancy.	United States	A total of 125 women with singleton pregnancies (<16 weeks), aged 18–40 years, BMI between 18.5 and 34.99 kg/m^2^, and no preexisting health conditions.	The effect of prenatal exercise on maternal and neonatal health to mitigate disparities between non-Hispanic Black women compared to non-Hispanic white women, in terms of a reduction in maternal and fetal negative outcomes.	Aerobic exercise 150 min/week vs. low-intensity activity	78%	Prenatal exercise reduced borderline significant racial/ethnic disparities in terms of the risk of preterm birth and gestational age with no effects found for the risk of cesarian section and neonatal birthweight.
Yew et al. [63]	Randomized controlled trial	Singapore	A total of 340 pregnant women diagnosed with GDM.	Effect of Habits-GDM, a smartphone application (app) coaching program, to prevent excessive GWG and to improve glycemic control and maternal and neonatal outcomes in GDM.	Standard care vs. integrated additional support through Habits-GDM app that integrated dietary, physical activity, weight, and glucose monitoring.	Difficult to evaluate	No statistically significant differences in the proportions of women with excessive GWG, absolute GWG, or maternal and delivery outcomes between experimental groups. Average glucose readings were lower in the intervention group.
Gilbert et al. [64]	Single-blind randomized controlled trial	Losanna	A total of 112 pregnant women with GDM.	The effect of a pre- and postpartum multidimensional interdisciplinary lifestyle and psychosocial interventions and their effects on maternal and offspring metabolic and psychobehavioral outcomes.	Usual guidelines-based care vs. personalized care focused on improving the diet, physical activity, and mental health of the mother until 1 year postpartum.	NA	There were no differences between groups in offspring birth, neonatal, anthropometric, or psychobehavioral outcomes up to one year. After adjustments for maternal age and the offspring’s sex and age, there was a borderline significant between-group difference in birth length, with the offspring of the mother from the intervention group being slightly shorter.
Chen et al. [65]	Randomized controlled trial	Taiwan	A total of 92 pregnant women <17 week of gestational age who were obese or overweight.	To examine the effects of the intervention of the mHealth app on overweigh/obese women during pregnancy.	Active healthcare with the mHealth app which helped women with a training schedule and dietary indications vs. standard care	NA	The intervention group’s newborns’ birthweight was significantly lower than that of the control group.
Dodd et al. [66]	Randomized controlled trial	Australia	A total of 970 pregnant women with a singleton gestation between 10 and 20 weeks who were overweight or obese.	The effect of providing antenatal dietary and lifestyle advice on neonatal anthropometry.	Standard care vs. dietary and lifestyle advice, including a combination of dietary, exercise, and behavioral strategies	NA	It was reported that there was a significant 18% reduction in the incidence of infants with birthweight >4.0 kg, and a 41% reduction in the incidence of birthweight >4.5 kg in the intervention group.

GA = gestational age; GDM = gestational diabetes mellitus; GWG = gestational weight gain; NA = not available.

**Table 2:** Summary of studies assessing the impact of maternal physical activity and lifestyle interventions during pregnancy on childhood BMI outcomes.

Author	Type of Study	Location	Sample	Study Purpose	Intervention Strategy	Adherence Rate (%)	Main Result
Dodd et al. [66]	Randomized controlled trial	Australia	1015	Effect of a dietary and lifestyle intervention compared with routine antenatal care for pregnant women with overweight and obesity on their children’ BMI at the age of 8–10 years old.	Standard care vs. dietary and lifestyle advice, including a combination of dietary, exercise, and behavioral strategies	NA	Dietary and lifestyle advice for women with overweight and obesity in pregnancy did not reduce the risk of childhood obesity, with children remaining at risk of adolescent and adult obesity.
Haby et al. [70]	Non-randomized controlled standardized trial	Sweden	Children of 538 pregnant women with obesity	The effect of intervention aimed at reducing GWG in women with obesity delivered during regular antenatal care in their children’s BMI at 2–5 years of age.	Standard care vs. standard care + lifestyle support via motivational talks with midwife and support from dietician.	Only 27% fulfilled the entire criteria of adherence	The mother’s BMI at the beginning of pregnancy is associated with the child’s BMI, but the lifestyle intervention performed for pregnant women with obesity could not demonstrate an effect on offspring’s BMI at 2.5 years of age, regardless of a reduced GWG in the intervention group.
Bjøntegaard [6]	Multicentric randomized controlled trial	Norway	A total of 855 pregnant women, among who only 281 participated to follow-up for 7 years.	The effect of regular moderate-intensity exercise during pregnancy on the children’s BMI at 7 years of age.	Exercise 3 times/week at moderate to high intensity and participation in at least one group session/week vs. standard prenatal care	During the intervention period, only 57% of women in the intervention group adhered to the exercise protocol.	Women adhering to the exercise protocol had significantly lower weight and BMI compared with the control group. However, it did not affect children’s BMI at 7 years. Children’s BMI is strictly correlated to their mothers’ BMI.
Perales [7]	Randomized controlled trial	Madrid (Spain)	A total of 1348 Caucasian women aged 18–45 years, free of contraindications for performing gestational exercise and with uncomplicated singleton pregnancies.	The effect of prenatal exercise on maternal and neonatal health as well as on children’s BMI at 1 year.	Supervised exercise 3 times/week vs. standard care	Adherence and exercise intensity is monitored through heart rate monitors	Pregnancy exercise slightly reduced the risk of macrosomia and of childhood overweight/obesity during the first year of life.

BMI = body mass index; NA = not available.

**Table 3:** Summary table of studies included on the impact of maternal exercise during breastfeeding on childhood obesity.

Author	Type of Study	Location	Sample	Study Purpose	Intervention Strategy	Adherence Rate (%)	Main Results
Aparicio VA et al. [81]	Pseudo randomized controlled trial	Spain	A total of 24 pregnant women from (exercise group) n = 23 (control group).	To examine the effect of an exercise intervention during pregnancy on inflammatory markers in both colostrum and mature HBM.	A total of 3 intervention groups: 3 times/week from the 17th week of gestation until delivery. Each session included 10 min of warm-up, 40 min of circuit training, 10 min of cool-down. Control group: standard care.	n.a.	Mothers on the exercise program exhibited 36% lower concentrations of IL-8 and 27% lower concentrations of TNF-α in their colostrum compared to the control group (*p* < 0.05 and *p* < 0.01, respectively). Mothers in the exercise program group showed a 30% increase in fractalkine levels (*p* = 0.05) and a borderline significant 20% higher concentration of IL-10 (*p* = 0.100) in mature milk.
Nguyen PTH et al. [82]	Prospective cohort study	Vietnam	A total of 1715 women between 24 and 28 weeks of gestation and followed until 12 months postpartum.	To assess the relationship between antenatal PA and the duration of breastfeeding.	n.a.	n.a.	Mothers with higher levels of PA exhibited lower risk of breastfeeding cessation after one year postpartum. Mothers with increased PA demonstrated higher rates of breastfeeding at 12 months compared to those with lower activity levels (odds ratio, OR = 1.71 vs. 1.38).
Be’er et al. [83]	Prospective, randomized, crossover clinical trial	Israel	A total of 31 healthy mothers during lactation.	To evaluate the impact of moderate-to-high-intensity PA on the volume and macronutrient composition of HBM.	Mothers followed 1 day of moderate-to-high-intensity aerobic PA and 1 day without PA. The intensity of PA was classified based on the RPE scale.		Moderate-to-high-intensity PA did not significantly affect the levels of macronutrients (fat, carbohydrates, protein) or the energy content of HBM. The volume of expressed milk remained unchanged.
Holmen M et al. [85]	Randomized cross-over study	Norway	A total of 20 women who exclusively breastfeed (6–12-week-old term infant).	To evaluate the effect of different PA intensities (MICT, HIIT) vs. rest (REST) on adiponectin concentrations in HBM.	Three conditions in the laboratory: REST (45 min), MICT (walking or jogging for 48 min), and HIIT (10 min warm-up at moderate intensity followed by four 4 min work-bouts, separated by 3 min of low-to-moderate intensity).	n.a.	A significant increase in adiponectin concentrations 1 h after HIIT, with a 0.9 µg/L change (95% CI: 0.3 to 1.5) compared to the REST condition (*p* = 0.025).
Biddulph C et al. [89]	Observational study	Australia	A total of 101 healthy women during lactation (3–4 months postpartum).	To investigate the potential effects of short-term maternal factors (including PA) on HMO profiles in mature HBM.	n.a.	n.a.	Maternal postpartum PA levels had a significant effect on HMO composition (*p* > 0.05).
Cabrera-Domínguez G et al. [92]	Cross-sectional study	Spain	A total of 60 women during lactation.	To evaluate maternal variables (e.g., quality of life, sleep hygiene, and PA) in women during both pregnancy and lactation.	n.a.	n.a.	Significant reduction in daily PA during the lactation period. A decrease in total PA was specifically associated with breastfeeding (β = 1683.67 ± 688.05; *p* = 0.016).

HBM = human breast milk; HMO = human milk oligosaccharides; HIIT = high-intensity interval training; MICT = moderate-intensity continuous training; n.a. = not available; PA = physical activity; REST = no activity.

**Table 4:** Summary table of relevant studies on the role of breastfeeding in controlling weight.

Author	Type of Study	Location	Sample	Study Purpose	Intervention Strategy	Main Results
Ong YY et al. [106]	Prospective cohort study	Singapore	A total of 839 children	Associations between the timing of the introduction of complementary foods, the duration of BF, and their interaction with child adiposity	Infant feeding practices data collection. Measurement of adiposity and cardiometabolic risk markers.	Children breastfed for less than four months had increased BMI at age 6 with respect to those breastfed for over four months
Hildebrand JS et al. [107]	Cross-sectional study	United States	A total of 823 children, ages 4–8 years	Breastfeeding associations with childhood obesity and body composition	Demographic and lifestyle information data collection. Infant feeding practices data collection. Infant height and weight data collection. Measurement of fat and fat-free body mass.	Dose–response relationship between breastfeeding and the likelihood of developing pediatric obesity—60% reduction in the risk for children breastfed for 6 months compared to non-breastfed
Rzehak P et al. [152]	Longitudinal study	Australia and Europe	A total of 6708 individuals	Identify growth patterns and investigate early nutritional programming potential on growth patterns BMI	Data collection on full breastfeeding, infants’ anthropometry, and body composition.	Exclusive breastfeeding was associated with a 1% decrease in BMI and a 2% reduction in fat mass at 6 years of age
Wijnands KP et al. [123]	Cross-sectional study	Netherlands	A total of 120 healthy children at 17 months of age	Investigate the influence of breastfeeding on infant DNA methylation levels	Data collection about infant conditions in the periconception, prenatal, and postnatal periods. Blood sample collection from mothers and children.	Negative correlation between the duration of breastfeeding and LEP gene methylation level
Sherwood et al. [124]	Comprehensive epigenome-wide association study	England	Children aged 10	Identify associations between breastfeeding and DNAm patterns in childhood	Data collection about infants’ allergic disease status, breastfeeding practice, and infant nutrition. Peripheral blood sample collection.	Decreased LEP gene methylation level in those who were breastfed for longer
Cheshmeh et al. [127]	Case–control study	Iran	A total of 150 healthy infants aged 5–6 months into 3 groups: breastfed, formula-fed, mix-fed.	Investigate the effect of breastfeeding, formula feeding, and mix feeding (breastfed plus formula-fed) on the expression level of FTO gene	Data collection about infant feeding practices. Complete children physical checkup. Blood sample collection.	FTO gene expression level was significantly lower in breastfed infants compared to formula-fed and mixed-fed infants.
Kanders et al. [130]	Cohort study	Sweden	A total of 4712 adolescents	To assess the moderating effect of breastfeeding duration on the relationship between FTO rs9939609 and BMI	Data collection through a self-reported questionnaire. Saliva sample collection.	Breastfeeding moderating action in the association between FTO rs9939609 polymorphism and child BMI
Wu YY et al. [131]	Longitudinal study	England	A total of 5590 children	Investigate exclusive breastfeeding role on child FTO gene expression and BMI trajectories	Parent and child data collection (FTO genotypes included). Questionnaire about routine healthcare and clinic attendance.	Five months of exclusive breastfeeding attenuated the increase in BMI and delayed the timing of the adiposity peak among FTO rs9939609 variant carriers
Dedoussis et al. [132]	Study that includes three independent pediatric cohorts (GENDAI, GENESIS, and ALSPAC study)	Greece and England	A total of 7837 children	Assess whether breastfeeding mediated the known association between FTO and BMI	Data collection about infants’ health and breastfeeding history. Blood and/or saliva sample collection.	Correlation between breastfeeding and rs9939609-rs17817449 FTO variants in 2 Greek cohorts, but not in the British cohort
Pauwels et al. [133]	Prospective, observational cohort study	Belgium	A total of 101 mothers and children	Assess the effect of breastfeeding duration on infant growth and methylation levels in obesity-related genes	Data collection about health status, socio-demographic factors, lifestyle habits, and physical activity of the mothers. Data collection about infants’ birthweight, length, delivery type, type and duration of feeding. Saliva samples collection.	Infants breastfed for 6 months had significantly lower RXRA gene methylation levels compared to non-breastfed infants
Verier et al. [134]	Cross-sectional study	Europe	A total of 945 adolescents (mean age 14.7 years)	Assess the modulating effect of breastfeeding on PPARG2 Pro12Ala polymorphism	Anthropometric measurements and physical activity data collection. Data collection about PPARG2 Pro12Ala polymorphism and breastfeeding. Data collection about weight and height at birth and duration of gestation. Blood samples collection.	BMI, waist circumference, and skinfold thickness all decreased with an increased duration of breastfeeding compared to children who were formula-fed
Lodemore et al. [146]	longitudinal study	England	A total of 26 infants from 6 to 16 weeks old	Influence of breastfeeding on circadian rhythmicity in body temperature	Weekly overnight recordings of infant body temperature from 6 to 16 weeks of age. Recordings about any episodes of illness in the infant. Basic perinatal, weight and height, feeding regimen data collection.	Circadian rhythmicity in body temperature appeared earlier in BF respect to formula-fed infants.

BF = breast feeding; BMI = body mass index; FTO = fat mass and obesity-associated protein; RXRA = Retinoid X Receptor Alpha; PPARG = peroxisome proliferator-activated receptor gamma.

**Table 5:** Main studies included on physical intervention strategies during pregnancy and breastfeeding.

Author	Study Type	Location	Sample Size	Study Purpose	Intervention Strategy	Adherence Rate (%)	Main Results
Hanley et al. [156]	Systematic review	England	2086 (n = 13 articles)	Determine if exercise could limit excessive gestational gain weight and reduce prolonged postpartum weight retention.	Exercise interventions included: swimming sessions; light-to-moderate aerobic exercise (intensities between 55 to 80%); resistance exercise; rate of perceived exertion between 10 and 14 (describing activities between very light to somewhat hard).	50 to 96% (50% adherence rate was found in a study that included exercise activities with intensities up to 80% of the HR)	Exercise was successful in reducing gestational weight gain during pregnancy in 45% of the studies included. Moderate-intensity exercise is affecting weight management.
Hawkins et al. [157]	Intervention study supervised remotely (RCT)	USA (Western Massachusetts)	260	Evaluate the effectiveness of an individually tailored, motivationally matched exercise intervention on physical activity levels in an ethnically diverse sample of pregnant women at high risk for gestational diabetes mellitus.	Self-selected activities chosen among specific activities suggested by the experimenters; Moderate-intensity activities, including dancing, walking, and yard work; Exercise protocols 10% weekly increases in activity time; Phone calls to check motivation levels and experienced barriers; Tailored advice and tips on how to overcome barriers.	41% of the women in the exercise group completed all the prescribed weekly sessions. Overall, 33% of post-intervention data were missing.	The group that performed exercise increased more in terms of their level of PA compared to the group involved in the PA promotion intervention.
Lewis et al. [158]	Telephone-based intervention study (RCT)	USA (Minnesota)	450	Investigate the efficacy of exercise and wellness interventions on preventing postpartum depression and perceived stress.	A total of 11 telephone sessions (1 weekly sessions during the first month and 2 weekly sessions during second and third months; 1 monthly session during the last three months) delivered by health professionals and trained educators; The suggested training intensities varied from 55 to 70%; Participants were asked to train a minimum of 30 min per day, divided in bouts of a minimum duration of 10 min.	88% adherence at 6-month follow-up; 87% adherence at 9-month follow-up.	Educating women about topics related to health and well-being helped them reduce stress and depression levels during the postpartum period. There was no support for the preventive role of exercise for postpartum depression; therefore, exercise alone may not be effective to prevent symptoms of depression.
Peralta et al. [159]	Systematic review on group-based interventions	Australia (Sydney)	779 (n = 6 articles)	Evaluate and summarize the findings of all relevant group-based PA studies to examine the effects on PA levels or other health behavior outcomes in postpartum women.	On-site attendance interventions Exercise protocols included yoga activities and combined exercise including moderate-intensity aerobic training and strengthening exercises; Group walks; Remotely supervised interventions PA advice included walking activities, strengthening, stretching, and flexibility; Mixed interventions including both center- and home-based protocols; Interventions included mother and newborn activities and counseling sessions delivered by educators and/or health professionals; All interventions consisted of exercise protocols varying between 20 min and 1 h of training.	100% only in one study lasting one month; 86 up to 96%.	The studies did not significantly increase the levels of PA in the 2 years after postpartum. However, interventions focusing mainly on PA promotion are more effective than multifaceted interventions on the achievement of minimum PA levels suggested by the WHO guidelines.

PA = physical activity; RCT = randomized controlled trial; HR = heart rate.

## Data Availability

Not applicable.
